# Role of circadian clock in female embryo implantation

**DOI:** 10.3389/fcell.2025.1607491

**Published:** 2025-06-04

**Authors:** Yukai Zhou, Xiaodie He, Zhenxin Chen, Yucong Gou, Kaixin Zhou, Jinrong Huang, Mei Chen, Jin Hong, Lei Gao

**Affiliations:** ^1^ State Key Laboratory of Plateau Ecology and Agriculture, Qinghai University, Xining, China; ^2^ College of Agriculture and Animal Husbandry, Qinghai University, Xining, China

**Keywords:** circadian clock, female reproduction, embryo implantation, reproductive hormone, implantation factor

## Abstract

Accumulating evidence indicates that circadian rhythm disruption can exert an impact on female reproductive function. In the context of female reproduction, the success of embryo implantation is of utmost significance as it is an essential process for female reproduction. Studies have demonstrated that females with disrupted circadian rhythms are more likely to experience embryo implantation failure, which is exemplified by shift workers, nurses, and flight attendants. Therefore, comprehending the circadian rhythm of female embryo implantation is crucial for human reproduction. Herein, we emphasize the mechanism of circadian operation and its regulatory effect on reproductive hormones related to embryo implantation. More importantly, the regulatory role of peripheral clock genes in the process of embryo implantation (endometrial receptivity and decidualization) is highlighted. Finally, melatonin is hypothesized to be a promising treatment for implantation failure caused by circadian rhythm disturbances.

## 1 Introduction

**FIGURE 1:**
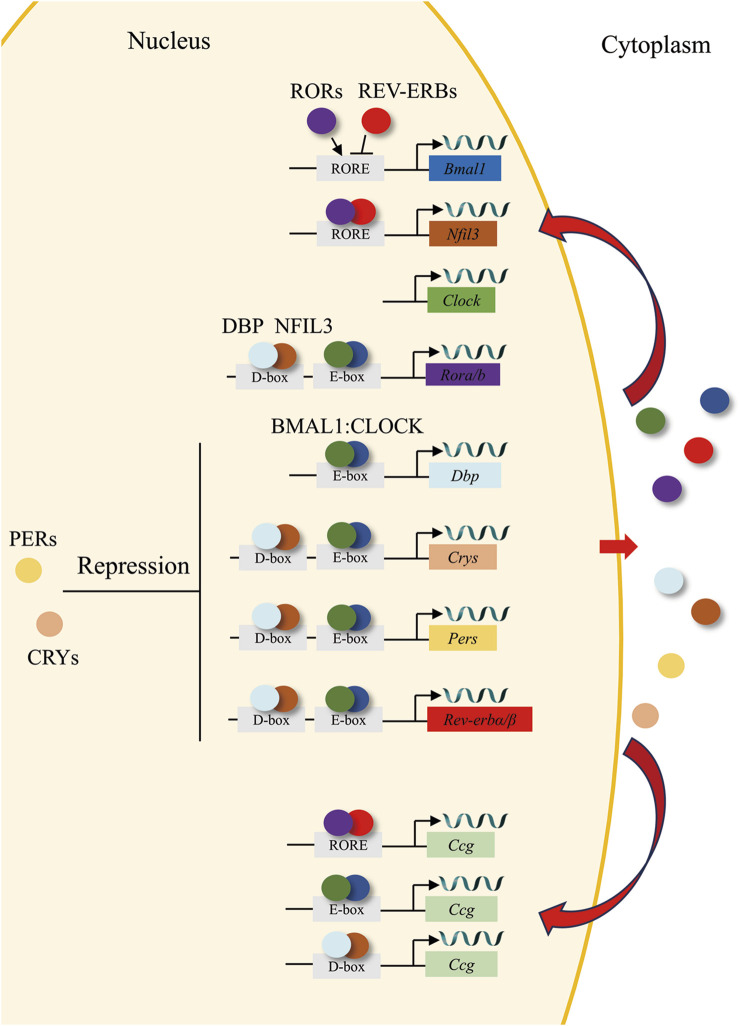
The circadian clock loop. In the core feedback loop, the transcription factors BMAL1 (blue circles) and CLOCK (green circles) bind to E-box domains on gene promoters, including the genes for *Rev-erbs* (red), *Pers* (yellow), and *Crys* (orange). PERs (yellow circles) and CRYs (orange circles) dimerize and translocate to the nucleus, where they repress their own, *Rors* (purple), and *Rev-erbs* transcription. In a second feedback loop, CLOCK and BMAL1 also regulate the transcription of genes for the nuclear receptors REV-ERBs (red circles), which compete with the retinoic acid-related orphan receptors, RORs (purple circles) for binding to RORE elements on the *Bmal1* gene promoter, providing both positive (ROR) and negative (REV-ERB) regulation of BMAL1 transcription. A third feedback loop is mediated by BMAL1: CLOCK-mediated transcription of the gene *Dbp* (cyan) and the ROR/REV-ERB-mediated transcription of *Nfil3* (brown). DBP (cyan circles) and NFIL3 (brown circles) dimerize and bind to D-box elements on the promoters of many of the core clock genes, providing additional layers of regulation. Ultimately, clock genes translocate to the nucleus to regulate the expression of CCGs.

The central circadian clock, situated in the suprachiasmatic nucleus (SCN) of the hypothalamus, functions as an autonomous pacemaker that synchronizes with the light-dark cycle and/or other temporal cues (zeitgebers) through retinal synaptic inputs and regulates physiological and behavioral rhythms to facilitate anticipation of predictable environmental changes. It is widely accepted that circadian rhythms that persist in the absence of zeitgebers are controlled by the central circadian clock (Bailey and Silver, 2014). The SCN contains a variety of neuronal subtypes, among which the expressing neurons of vasoactive intestinal peptide (VIP), arginine vasopressin (AVP), and neuromedin-S (NMS) exhibit strong circadian gene expression (Lee et al., 2015; Wen et al., 2020). The SCN is trained by environmental signals and conveys the external periodicity to the peripheral clocks in the remaining parts of the body. External environmental factors, including light–dark cycles, temperature, feeding times, and physical activity, function as circadian time cues, or zeitgebers, to generate endogenous rhythms with a period approximately close to 24 h. Light is regarded as the primary zeitgeber that imparts timing to the endogenous clock and facilitates the process through which an individual’s internal period is adjusted to align with that of its environment (Takahashi, 2017; Cox and Takahashi, 2019). In mammals, the core of the cellular and molecular clock mechanism is composed of transcriptional activators such as brain and muscle arnt-like protein 1 (BMAL1) and circadian locomotor output cycle kaput (CLOCK). These two elements form a heterodimer (BMAL1: CLOCK) and attach to the enhancer box (E-box), which possesses the DNA sequence CANNTG (where 'N' stands for any nucleotide) within the promoter area of both target clock genes and clock-controlled genes. The target clock genes mainly comprise, but are not limited to, the period (*Per1-3*) and cryptochrome (*Cry1-2*) genes (Shearman et al., 1997; Kume et al., 1999). The PER and CRY proteins undergo post-translational modifications and subsequently return to the nucleus. There, they function as cyclic repressors for the transcription of their own genes and other related genes by interfering with the binding of the BMAL1: CLOCK complex to the DNA (Michael et al., 2017; Rosensweig et al., 2018). In the secondary major transcriptional loop, BMAL1: CLOCK initiate the transcription of genes encoding the nuclear receptors REV-ERBα and REV-ERBβ (Preitner et al., 2002). These proteins contend with the retinoic acid-related orphan receptors, namely, RORα, RORβ, and RORγ, for the binding sites ROR-binding elements (RORE) on the BMAL1 gene. This interaction leads to both positive (ROR) and negative (REV-ERB) transcriptional regulation (Sato et al., 2004). A tertiary feedback loop encompasses the D-box binding protein (DBP) and the nuclear factor, interleukin-3 regulated protein (NFIL3 or E4BP4). These are regulated by BMAL1: CLOCK and CRY1, and they attach to D-box elements on circadian promoters, such as those of RORα and RORβ (Ueda et al., 2005; Ripperger and Schibler, 2006; Stratmann et al., 2010). These interconnected feedback loops collectively constitute the “molecular clock”, governed by transcriptional-translational mechanisms and exhibiting a self-sustained circadian oscillation period approximating 24 h (Takahashi, 2017; Cox and Takahashi, 2019). The core clock gene also drives the expression of clock-controlled genes (CCGs), including embryo attachment-related genes (Figure 1), which act as cell- and tissue-specific regulators of rhythmic physiological function.

In females of mammalian species, reproductive activity exhibits regular cyclic patterns, namely, menstrual cycles in women and estrous cycles in other mammals. These cycles are orchestrated by intricate interactions among hypothalamic neuropeptides, pituitary gonadotropins such as luteinizing hormone (LH) and follicle-stimulating hormone (FSH), sex steroid hormones secreted by the ovaries, and the circadian system (Simonneaux and Bahougne, 2015). The ultimate result of this regulatory mechanism is to synchronize the production of ovulation after oocyte maturation with the reproductive tract of the recipient area, thus ensuring the normal development of the embryo (Simonneaux and Bahougne, 2015). Studies focusing on SCN damage in rodents have indicated that such damage induces a diverse array of reproductive dysfunctions, manifested as disruptions in the estrous cycle, aberrations in follicular development, and perturbations within the ovulatory reproductive process (Silva et al., 2023; Vieyra et al., 2024). In humans, circadian disruption has been established to have an association with reproductive dysfunction and subfertility. In circadian rhythm disruption scenarios such as shift work, women are more predisposed to report irregular menstrual cycles (Baker and Driver, 2007). Chronic circadian disruption in humans correlates with augmented pregnancy latency and a higher incidence of miscarriage, with the maximal risk manifesting during early pregnancy. Prolonged engagement in shift work has also been correlated with an elevated risk of preterm birth and the occurrence of infants with low birth weight (Baker and Driver, 2007; Sen and Sellix, 2016).

In mammals, a novel life commences with the fusion of an ovum and a sperm, which is termed fertilization. After this event, the zygote experiences multiple rounds of division and morphogenetic processes, ultimately giving rise to the blastocyst. The blastocyst represents an embryonic stage that comprises two distinguishable cell lineages: the outer trophectodermal epithelium with specialized characteristics and the inner cell mass (Wang and Dey, 2006; Cockburn and Rossant, 2010). Successful implantation requires synchronization between the acquisition of implantation competency by the blastocyst and a receptive state in the uterine endometrium (Dey et al., 2004; Wang and Dey, 2006). These two events are precisely regulated by maternal hormones, in particular, ovarian estrogen and progesterone (Conneely et al., 2002; Cheng et al., 2023). Molecular and genetic evidence indicates that ovarian hormones together with locally produced signaling molecules, including cytokines, growth factors, homeobox transcription factors, lipid mediators and morphogen genes, function through autocrine, paracrine and juxtacrine interactions to specify the complex process of implantation (Dey et al., 2004). The crosstalk between the blastocyst and the uterus is restricted to a short period, termed the “window of implantation” (Paria et al., 1993; Ma et al., 2003). Upon encountering the implanting embryo, the adjacent uterine stroma undergoes a cellular transformation process known as decidualization, which is essential for facilitating embryonic growth and invasion (Lim and Wang, 2010). The locally formed decidua provides a positive feedback mechanism that promotes embryo survival. Any disruptions in this process can lead to unfavorable consequences for subsequent developmental events such as decidualization and placentation, and may even result in the termination of the pregnancy (Ye et al., 2005; Chen et al., 2011). Research investigations into the reproductive capacities of female shift workers (engaged in work between 18:00 and 7:00), nurses, and flight attendants have all found that women with disrupted circadian rhythms have a higher incidence in terms of menstrual disorders, infertility, and pregnancy failures (Zhu et al., 2003; Quansah and Jaakkola, 2010; Grajewski et al., 2015). The preponderant majority of pregnancy failures stem from the failure of embryo implantation (Wilcox et al., 1988). Hence, it is of utmost importance to explore the role of circadian rhythm in embryo implantation and to tackle this global issue. This review will examine our understanding of circadian regulation of embryo implantation.

## 2 Reproductive hormones regulated by the circadian clock are involved in embryo implantation

**FIGURE 2:**
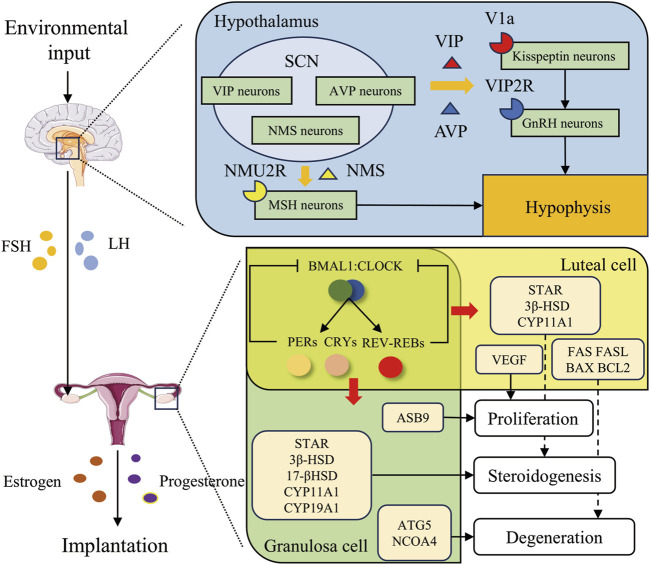
Circadian rhythms regulate embryo implantation through reproductive hormones. The suprachiasmatic nucleus, situated within the hypothalamus, is susceptible to environmental stimuli. It exerts regulatory effects in two main aspects. Firstly, it prompts the secretion of GnRH and the pituitary to release FSH and LH, thereby modulating the ovarian secretion of estrogen and progesterone. Secondly, it influences the secretion of estrogen and progesterone through the peripheral circadian clock loop. Ultimately, these processes facilitate embryo implantation. Specifically, in the hypothalamus, VIP, AVP, and NMS secreted by time-controlled neurons within the SCN regulate gonadotropin secretion through binding to V1a receptors on Kisspeptin neurons, VIP2R receptors on GnRH neurons, and NMU2R receptors on MSH neurons, respectively. In the ovary, clock genes modulate the secretion of estrogen and progesterone by regulating genes associated with proliferation, steroidogenesis, and degeneration in granulosa and luteal cells.

Studies have shown that estrogen (E_2_) and progesterone (P_4_) secreted by the ovary play a critical regulatory role in the process of embryo implantation (Sandra, 2016). The synergistic effect of these two hormones promotes the establishment of uterine receptive state, which is conducive to the occurrence of embryo implantation (Sandra, 2016). Based on the dynamic fluctuation patterns of E_2_ and P_4_ during embryo implantation, Finn and Martin classified it into three processes. The details of these three processes, supplemented with data from subsequent research, are as follows. In the first stage, LH and FSH induce an elevation in E_2_ levels, which stimulates follicular development and subsequently leads to ovulation. Subsequently, P_4_ levels experience a continuous increase concomitant with the production of corpus luteum and then return to normal levels. The second stage is characterized by relatively low levels of both hormones. The third stage is marked by the occurrence of mating behavior (Finn and Martin, 1974). Implantation takes place at the end of the third stage, during which P_4_ secretion continuously rises, reaches a peak, and sustains peak secretion. Notably, in the third stage, a transient and relatively small E_2_ peak emerges on the first day of implantation, which is associated with the release of delayed implantation (Finn and Martin, 1974; Paria et al., 1993; Zhang et al., 2013). In the pre-implantation period, if E_2_ supply to the uterus is interrupted, the blastocyst fails to implant, and the uterus enters a state of delayed implantation. P_4_ supplementation alone does not reverse this condition. Nevertheless, the administration of exogenous E_2_ can disrupt this state and trigger blastocyst implantation (Paria et al., 1993). Furthermore, E_2_ modulates uterine receptivity and decidualization via E_2_ receptors α and β (Winuthayanon et al., 2010; Pawar et al., 2015). Although E_2_ is crucial in the embryo implantation process, P_4_ has emerged as the most essential hormone for successful implantation owing to its specific properties. The nuclear receptors P_4_ receptors (PR) A and B are expressed in the uterus. It is postulated that PRA is implicated in embryo attachment. Both global PRA knockout mice and uterus-specific knockout mice exhibit infertile phenotypes (Conneely et al., 2001; Lee et al., 2006). A substantial body of research has demonstrated that P_4_/PRA signaling restrains the proliferation of epithelial cells. Additionally, it promotes stromal cell proliferation and differentiation by activating multiple downstream signaling molecules. Consequently, this signaling pathway facilitates the establishment of uterine receptivity as well as the occurrence of embryo implantation and decidualization (Conneely et al., 2001; Lee et al., 2006) Reproductive hormones play a pivotal role in embryo attachment. Intriguingly, as shown in Figure 2, these hormones are regulated by the circadian rhythm.

### 2.1 The circadian clock regulates gonadotropins

The circadian regulation of LH by the hypothalamic-pituitary-gonadal (HPG) axis serves as a key signal for corpus luteum formation and ovulation, as shown in Figure 2, which is essential for the proper progression and coordination of reproductive processes. In the hypothalamus, kisspeptin neurons stimulate Gonadotropin-releasing hormone (GnRH) neurons, which release GnRH onto gonadotropes in the anterior pituitary. In response to GnRH, gonadotropes release LH and FSH into the circulation, allowing these hormones to act on the gonads to stimulate gametogenesis and sex steroid production. When the follicles are mature, the level of estrogen released reaches a threshold level, which then becomes an activator to kisspeptin neurons in the anteroventral periventricular nucleus (AVPV). The increased activity and release of kisspeptin by AVPV kisspeptin neurons onto GnRH neurons results in a surge of GnRH, which prompts a surge of LH, and then ovulation (Wang and Moenter, 2020). The VIP acceptor two is expressed on GnRH neurons and VIP neurons located in the SCN possess the capacity to project directly onto GnRH neurons (Van der Beek et al., 1994; An et al., 2011). The absence of VIP leads to a reduction and delay in the LH surge, consequently resulting in impaired ovulation and reduced fertility in mice (Harney et al., 1996; Loh et al., 2014; Hoffmann et al., 2021). AVP neurons in the SCN shell project to AVPV kisspeptin neurons in rodents by vasopressin receptor 1a (V1a), and AVP robustly stimulates kisspeptin neuron firing (Williams et al., 2011; Piet et al., 2015). In SCN-lesioned animals, intracranial injection of AVP in the late afternoon rescues the LH surge through V1a (Palm et al., 1999; Miller et al., 2006). Neuromedin U receptor type 2 (NMU2R), the receptor for NMS, is widely expressed in the hypothalamus and anterior pituitary, particularly in melanocyte-stimulating hormone (MSH) neurons (Crown et al., 2007; Yang et al., 2010). Evidence suggests that NMS regulates luteinizing hormone (LH) secretion by acting on MSH neurons in pigs. Additionally, the administration of exogenous NMS increases serum LH levels in female rats, further supporting the regulatory role of NMS in LH secretion (Vigo et al., 2007; Yang et al., 2010).

### 2.2 Estrogen is secreted under the control of the circadian clock

E_2_ is the main hormone secreted by ovarian granulosa cells and plays an important role in embryo implantation. The steroidogenic acute regulatory protein (STAR) promotes the transport of cholesterol from the outside to the inside of the mitochondrial membrane. Under the catalysis of cytochrome P450 family 11 subfamily a member 1 (CYP11A1), cholesterol undergoes a side-chain cleavage reaction to generate pregnenolone. Pregnenolone is then converted into dehydroepiandrosterone (DHEA) under the action of 17-hydroxylase (CYP17A1). DHEA is catalyzed by 3β-hydroxysteroid dehydrogenase (3β-HSD) to produce androstenedione. Androstenedione is further converted into estrone under the action of aromatase cytochrome P450 family 19 subfamily a member 1 (CYP19A1). Estrone can be further transformed into estradiol with stronger activity under the action of 17β-hydroxysteroid dehydrogenase (17β-HSD) (Wallach et al., 1996). Accumulating evidence indicates that the circadian clock exerts regulatory control over E_2_ signaling. The knockdown of Clock genes *Bmal1* or *Clock* via small interfering RNA led to a reduction in the expression of *StAR*, *Cyp11a1*, and *Cyp19a1*, accompanied by a decrease in E_2_ content within granulosa cells. Conversely, the knockdown of *Per2* enhanced *StAR* expression and augmented E_2_ production. This may be the reason why PER2 is a BMAL1: CLOCK repressor (Shimizu et al., 2011; Wang et al., 2017). REV-ERBα further diminished estrogen secretion in ovarian granulosa cells through a direct interaction with the RORE region of the *Cyp19a1* promoter, which in turn suppressed *Cyp19a1* expression. Simultaneously, REV-ERBα could also act on the RORE region of the *Bmal1* promoter to curtail its expression and undermine *Bmal1* function, consequently leading to a reduction in E_2_ secretion (Cho et al., 2012; Wang et al., 2022). E_2_ is primarily secreted by ovarian granulosa cells (GCs), and the proliferation, apoptosis and autophagic processes of GCs can influence E_2_ production. A study utilizing RNA-seq analysis on GCs with CLOCK overexpression successfully identified Ankyrin repeat and suppressor of cytokine signaling box-containing 9 (ASB9) as a differentially expressed gene, which is involved in cellular growth and differentiation processes (Benoit et al., 2019; Huang et al., 2023). Experimental findings demonstrated that ASB9 is a direct target gene of CLOCK, through which CLOCK increases the population of cells in the G1 phase, reduces the number of cells in the G2 phase, and suppresses the viability of GCs (Huang et al., 2023). Circadian rhythms are not only involved in the proliferation of GCs but also play a role in GCs apoptosis. In an experiment involving the knockdown of *Bmal1* in porcine GCs, the phosphoinositide 3-kinase (PI3K)/protein kinase b (Akt)/mechanistic target of rapamycin (mTOR) signaling pathway was inactivated, indicating the onset of apoptosis in GCs. This finding was further corroborated by flow cytometry analysis (Wang et al., 2017). Autophagy, a tightly regulated lysosomal degradation pathway, is essential for clearing long-lived proteins and damaged organelles. Dysregulation of this process can have severe cellular consequences (Klionsky and Emr, 2000; Kroemer et al., 2010). Autophagy-related 5 (Atg5) is a CCG regulated by *Rev-erbα*, which negatively modulates *Atg5* expression, leading to autophagy dysregulation in mice GCs (Zhang et al., 2022). Nuclear receptor coactivator 4 (NCOA4) is a cargo receptor responsible for autophagy-dependent ferritin degradation (Mancias et al., 2014). NCOA4-mediated ferritinophagy maintains intracellular iron homeostasis by facilitating ferritin iron storage or release according to demand. NCOA4 deletion inhibits ferroptosis by blocking ferritinophagy and ferritin degradation (Liu et al., 2020). In human ovarian GCs, *Cry1* modulates NCOA4-mediated ferritinophagy by regulating NCOA4 ubiquitination and subsequent degradation. Furthermore, treatment with KL201, a *Cry1* stabilizer, effectively suppresses ferritinophagy (Ma et al., 2024). In summary, as synthesized in Table 1, the circadian system plays a fundamental role in regulating both the initiation and termination of ovarian GCs functions, critically influencing their capacity to secrete E_2_.

**TABLE 1:** Circadian coordination of reproductive hormone in embryo implantation.

Reproductive hormones	Produce	The regulatory role of circadian clock	Roles in implantation
Estrogen	Ovary	Ovarian apoptosis and estrogen synthesis	Receptivity, decidualization
Progesterone	Corpus luteum	Corpus luteum formation, regression, and progesterone synthesis	

### 2.3 The circadian clock regulates progesterone secretion and the luteal cycle

P_4_ is the main hormone secreted by the corpus luteum (CL). The source of cholesterol for steroidogenesis in ovarian luteal cells depends on circulating plasma lipoproteins, *de novo* synthesis, and utilization of intracellular cholesterol ester stores. STAR facilitates the transport of cholesterol from the outer to the inner mitochondrial membrane, serving as the rate-limiting step in progesterone synthesis. CYP11A1 catalyzes the conversion of cholesterol to pregnenolone, which passes into the smooth endoplasmic reticulum where it is converted to progesterone by 3β-HSD. P_4_ then diffuses out of the luteal cell to be transported to the target tissues (Yakin et al., 2023). Accumulating evidence suggests that the circadian clock exerts significant regulatory control over P_4_ signaling pathways. In an experiment involving mice subjected to constant light, the researchers discovered that mice with circadian rhythm disruptions induced by continuous light exposure exhibited lower *StAR* and serum P_4_ levels (Li et al., 2023). Continuous light exposure and a 6-h phase shift every 3 days of light exposure led to a reduction in serum P_4_ levels in ruminants (Gao et al., 2016; Suarez-Trujillo et al., 2022). Specifically, it is the peripheral clock proteins, such as BMAL1, that are operative. BMAL1 global knockout female mice were found to be infertile (Ratajczak et al., 2009; Boden et al., 2010). However, when additional P_4_ was continuously administered from day 3.5 to day 6.3 post-fertilization, embryo implantation and pregnancy establishment occurred. During pregnancy, at day 3.5, BMAL1 global knockout mice exhibited lower levels of *StAR* and serum P_4_ (Ratajczak et al., 2009). To validate the role of BMAL1 in the modulation of progesterone secretion within the ovary, Liu has ascertained this role through the specific knockout of BMAL1 in steroidogenic cells and subsequent ovarian transplantation (Liu et al., 2014).

In addition, as delineated in Figure 2, circadian rhythms also regulate luteinization and luteolysis. Luteinization is the basis for the secretion of progesterone by the corpus luteum. Ovulation leads to the establishment of a local hypoxic microenvironment. This hypoxic condition triggers a significant upsurge in hypoxia-inducible factor 1α (HIF-1α) (Zhang et al., 2011). Subsequently, HIF-1α associates with HIF-1β to form a heterodimer (HIF-1). The formed dimer then binds to the cis-hypoxia response element (HRE) located within the VEGF promoter region, thereby facilitating and enhancing VEGF mRNA expression (Kazi et al., 2005). The circadian-expressed CLOCK and PER2 functions as an effector molecule, which is involved in promoting the recruitment of HIF-1 to the HRE region of the VEGF promoter (Tang et al., 2015; Kobayashi et al., 2017). However, in zebrafish, PER2 was shown to inhibit VEGF (Jensen et al., 2012). In nucleus pulposus cells, the suppression of BMAL1 and RORα leads to the decrease of the expression of HIF-1 and VEGF (Suyama et al., 2016). BMAL1 even more has been demonstrated to act as a transcription factor in facilitating VEGF expression (Jensen et al., 2012; Guo et al., 2021; Zhang et al., 2023). The inhibitory proteins PER and CRY heterodimerize into the nucleus and directly interact with BMAL: CLOCK to inhibit its transcriptional function (Michael et al., 2017; Rosensweig et al., 2018). The functions of BMAL1 and PER2 are thus in opposition, and we are confident that PER2 regulates the role of HIF-1, but the role of PER2 in the regulation of VEGF is debatable. It is routinely assumed that PER2 regulates VEGF expression through HIF-1, but due to the inhibitory effect of PER2 on BMAL1, the pro-VEGF expression of BMAL1 is weakened, and the end result is attenuated VEGF (Koyanagi et al., 2003; Su et al., 2017). This indicates that *Vegf* is a CCG, and several studies have also corroborated this notion (Frigato et al., 2009; Wharfe et al., 2011; Yang et al., 2015). Although research on the regulation of VEGF by circadian genes during ovarian luteinization is currently lacking, the significance of circadian regulation of VEGF has been established by numerous investigations. Additionally, there is evidence indicating that BMAL1 coincides with the HIF-1 peak during luteinization (Kobayashi et al., 2018). This evidence suggests that circadian genes play a crucial role in modulating angiogenesis during luteinization. Luteal regression is essential in triggering the development of a new follicle and restarting the estrous cycle. During the stage of diestrus, the CL regresses, losing its capacity to produce P_4_ and under goes structural involution. Proapoptotic and antiapoptotic factors have been implicated in structural luteal regression. The pro-apoptotic factors, namely, *Fas*, *FasL*, and *Bax*, as well as the anti-apoptotic factor *Bcl-2*, are rhythmically expressed within the ovary. Additionally, the promoter regions of these factors all possess BMAL1-binding E-box sequences. During luteal phase apoptosis, the peak expression of these factors followed the peak in BMAL1 expression (De La Vega et al., 2018).

E_2_ and P_4_ regulated by the HPG axis, play crucial roles in endometrial receptivity and decidualization during embryo implantation. As shown in Table 1, these hormones are essential for the implantation process. Furthermore, they are themselves regulated by circadian rhythms, thereby mediating the circadian coordination of embryo implantation through this bidirectional regulatory mechanism.

## 3 The circadian clock is involved in embryo implantation and decidualization

**FIGURE 3:**
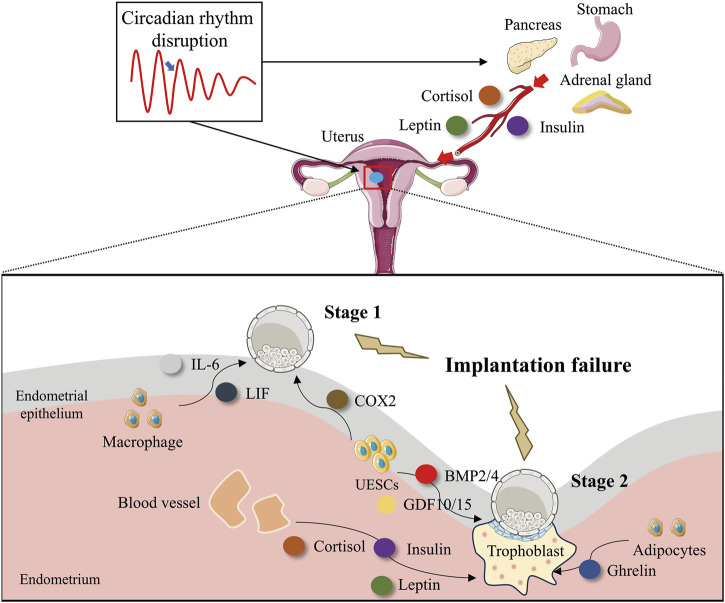
Circadian rhythm disruption impairs embryo implantation by interfering with implantation-associated factors. Disruption of circadian rhythms impairs embryo implantation by altering the secretion of key implantation factors. Disruption of circadian rhythms interferes with the normal temporal regulation of uterine-derived implantation factors, leading to dysregulated secretion patterns. This disturbance affects: Stage 1 (Uterine receptivity and blastocyst adhesion): Macrophage-derived IL-6 and LIF, as well as endometrial epithelial cell-secreted COX-2, which are critical for embryo attachment. Stage 2 (Decidualization and trophoblast invasion): Endometrial epithelial cell-derived BMP2/4 and GDF10/15, along with adipocyte-secreted leptin, which support stromal decidualization and placental development. Additionally, circadian misalignment directly disrupts the rhythmic secretion of cortisol (adrenal gland), insulin (pancreas), and ghrelin (stomach). These endocrine factors, upon reaching the endometrium via systemic circulation, further impair Stage 2 implantation processes, including decidual transformation and trophoblast function. This systemic dysregulation highlights the critical role of circadian homeostasis in successful embryo implantation.

In all eutherian mammals that have been investigated so far, the uterus undergoes a transformation into a modified state when blastocysts can engage in effective two-way communication to commence the implantation process. This state is designated as uterine receptivity for implantation and endures for a restricted time frame (Paria et al., 1993). During this period, the uterine milieu is capable of facilitating blastocyst growth, attachment, and the ensuing implantation procedures. Besides the E_2_ and P_4_ mentioned previously, as synthesized in Figure 3, multiple factors contribute to the determination of uterine receptivity (Dey et al., 2004). This stage is correlated with circadian rhythms. In a study of human endometrial RNA sequencing conducted prior to and during embryo implantation, significant discrepancies were identified in circadian pathway genes, indicating that circadian genes play an essential regulatory role in the embryo implantation process (Hu et al., 2014). Decidualization of the endometrium is a process involving a series of morphological and functional changes that occur in uterine endometrial stromal cells (UESCs) during embryo implantation. It is a crucial step for embryo implantation and maintenance of pregnancy, mainly manifested as the proliferation and differentiation of UESCs, as well as the remodeling of the extracellular matrix. Decidualization is subject to circadian regulation. It has been demonstrated that the peripheral clock systems play significant and essential roles in the process of decidualization (Muter et al., 2015; Lv et al., 2019; Zhang et al., 2019; Lužná et al., 2021).

### 3.1 Growth factors

**FIGURE 4:**
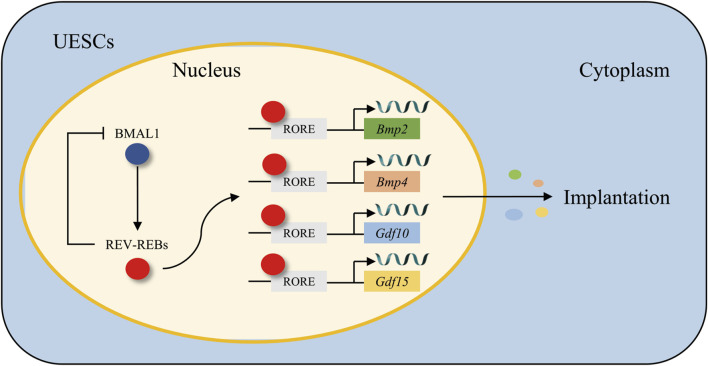
The regulatory mechanism of the BMAL1/REV-REBs loop in the modulation of growth factors *Bmp2/4* and *Gdf10/15*. BMAL1 transcriptionally activates REV-ERBs, which in turn binds to the RORE motifs in the promoters of *Bmp2/4* and *Gdf10/15*, forming a feedback loop that governs their transcriptional expression in UESCs, thereby synchronizing growth factor dynamics with circadian rhythms during embryo implantation.

Bone morphogenetic proteins (BMPs), which belong to the transforming growth factor-*β* (TGF-*β*) superfamily, are implicated in a diverse range of cellular functions, such as proliferation, differentiation, and remodeling (Shimasaki et al., 2004). The BMP family, comprising BMP2, BMP4, BMP6, and BMP7, exhibits spatiotemporal expression in the mouse uterus during the successive phases of implantation. BMP2 is abundantly expressed within the decidual area encircling the site of blastocyst attachment and assumes a crucial function in decidualization (Ying and Zhao, 2000; Li et al., 2007). Emerging evidence indicates that the circadian clock exerts stable and significant regulatory effects on BMPs. In both humans and rodents, UESCs undergo proliferation and differentiation into decidual cells. *In vitro* decidualization was induced by medroxyprogesterone acetate and 2-O-dibutyryl cAMP (He et al., 2007). It was observed that the knockdown of *Bmal1* led to a downregulation of *Rev-erbα* expression and an upregulation of *Bmp2/4/6* expression. Subsequent to the application of a REV-ERBα antagonist, the expression of *Bmp1/2/4/6/7/8a* was enhanced. These findings imply that *Rev-erbα* is a significant circadian clock gene that governs the BMP family and, as shown in Figure 4, functions as a transcription factor by binding to the RORE regions of the *Bmp2* and *Bmp4* promoters to modulate their expression (Tasaki et al., 2015).

Growth/differentiation factors (GDFs) are members of the TGF-*β* superfamily, and they are involved in a variety of cellular functions and biological processes such as cell proliferation, differentiation, and remodeling (Whitman, 1998). *Gdf10* and *Gdf15* are ubiquitously expressed throughout the uterus, especially during the crucial period of embryo implantation. This widespread expression pattern strongly suggests their significant and active roles in the implantation process (Fairlie et al., 1999; Zhao et al., 1999). Notably, both *Gdf10* and *Gdf15* exhibit a remarkable and significant increase in expression levels during the decidualization of UESCs, thereby further implying their essential contributions to the decidualization process. The circadian clock exhibits robust regulatory control over GDFs. When decidualized UESCs are treated with a REV-ERBα inhibitor, it leads to an upregulation in the expression of both *Gdf10* and *Gdf15*. Moreover, through further chromatin immunoprecipitation analysis, it has been revealed that REV-ERBα, as demonstrated in Figure 4, exerts its inhibitory effect on *Gdf10* and *Gdf15* by directly binding to their respective promoters (Zhao et al., 2016).

### 3.2 Prostaglandins

Prostaglandins (PGs) are produced through the hydrolysis of membrane phospholipids by cytoplasmic phospholipase A2 to release arachidonic acid, which is converted to PGs by Cyclooxygenase-2 (COX2) and PG endoperoxide H synthase. PGs intermediate the functions of the corpus luteum, participate in maternal–fetal interface immunomodulation and pregnancy identification, and stimulate angiogenesis during early pregnancy (Ye et al., 2021). PGs can also regulate myometrium relaxation and contraction via PG transporters and receptors, thus affecting blastocyst transportation and adhesion reactions of the endometrium–trophoblast, ultimately regulating the distribution of the implanted embryos in the uterus (Blitek and Szymanska, 2020). COX serves as the rate-limiting enzyme in the synthesis of PGs. Mice lacking COX-2 display unsuccessful embryo implantation and defective uterine decidualization (Lim et al., 1997). COX-2 is also a time-regulated gene, exhibiting robust rhythmicity at rat D3.5-4.5 of pregnancy. The suppression of *Bmal1* expression in rat UESC led to a reduction in the expression levels of *Cox-2* and PGE2. It was highly expected that (Figure 3) the inhibition of REV-ERBα, which is a repressor of *Bmal1*, would enhance the expression of *Cox-2* (Chen et al., 2013; Isayama et al., 2014; 2015; Zhao et al., 2021). This effectively illustrates the precise and effective regulation of the prostaglandin synthesis pathway by the circadian clock loop.

### 3.3 Cytokines

It is widely acknowledged that the interleukin (IL)-6 family, a group of cytokines, holds significant importance during embryonic implantation (Dimitriadis et al., 2005). The IL-6 family encompasses several cytokines, such as leukemia inhibitory factor (LIF), IL-6, IL-11, and neurotrophic factor. Among the cytokines that have been investigated, LIF is most relevant to implantation (Kimber, 2005; White et al., 2007). The expression of LIF exhibits a biphasic pattern on day 4, initially appearing in the uterine glands and subsequently in the stromal cells surrounding the blastocyst during the attachment reaction (Song et al., 2000; Ni et al., 2002). This expression profile implies that LIF has dual functions, being involved in uterine preparation initially and then in the attachment reaction (Stewart et al., 1992; Song et al., 2000). Female mice with a deficiency in LIF experience implantation failure, and this defect can be rescued by supplementation with LIF (Stewart et al., 1992). In addition to LIF, IL-6 is another crucial cytokine for successful pregnancy. In mice, it is secreted by the epithelial and stromal cells in the uterus and is regulated by ovarian steroid hormones (Prins et al., 2012). Mice lacking IL-6 display impaired implantation and a delayed onset of labor, leading to adverse pregnancy outcomes (Robertson et al., 2010). In humans, IL-6 is mainly produced by endometrial epithelium and stromal cells in a cyclic manner. The levels of IL-6 are relatively low during the proliferative phase and increase steadily during the secretory phase, suggesting its important role during implantation (Jasper et al., 2007; Champion et al., 2012). Melatonin (MT) represents a clock control hormone that is secreted by the pineal gland, ovary, and placenta and plays a crucial role in modulating endometrial receptivity and immunity (Cajochen et al., 2003; Wu et al., 2017). MT exerts its effects via two receptors, MT1/2, which are expressed in a circadian manner (Wu et al., 2017). In a research study on human endometrial receptivity, it was proposed that MT could enhance endometrial receptivity through the nuclear factor kappa B (NF-κB) and apoptotic pathways. Concurrently, MT also stimulated the expression of LIF and IL-6 in the endometrium (Guan et al., 2022; Zheng, 2022).

Studies have revealed that the internal time-keeping system circadian clock genes are responsible for driving the circadian rhythms evident in the immune system. For instance, the recruitment of immune cells (such as monocytes, neutrophils, and lymphocytes), antigen presentation, lymphocyte proliferation, and cytokine gene expressions occur in accordance with a 24-h daily rhythm, thereby initiating an acute response to infection (Nakao, 2014). In mouse aortic endothelial cells, the knockdown of the *Clock* gene led to a significant downregulation of LIF expression (Jiang et al., 2018). In mice with specific deletion of BMAL1 in myeloid cells, the temporal variations in serum IL-6 following lipopolysaccharide (LPS) challenge were not observed. BMAL1 exerts a downstream effect by activating the transcription of the nuclear receptor *Rev-erbα*, and REV-ERBα, in turn, inhibits BMAL1. Consequently, in *Rev-erbα*-deficient mice, these rhythmic immune responses to LPS were abolished. This finding implies that there is a connection among BMAL1, REV-ERBα, and the production of IL-6 in macrophages upon LPS challenge (Gibbs et al., 2012). This finding (Figure 3) is buttressed by the observed inhibition of IL-6 expression by REV-ERBα in bovine endometrial epithelial cells (Yang W. et al., 2024). Finally, *Cry* is also involved in uterine receptive immunity. Macrophages derived from *Cry1/2* knockout mice exhibited an enhanced secretion of IL-6 (Narasimamurthy et al., 2012).

### 3.4 Cortisol

The glucocorticoid hormone cortisol is a primary product of the hypothalamic-pituitary-adrenal (HPA) axis, a key biological stress response system. The effects of glucocorticoids are mediated by the glucocorticoid receptor (GR), which translocates to the nucleus in a ligand-dependent manner and acts as a transcription factor to regulate gene expression (Kumar and Thompson, 2005). Alterations in cortisol levels have been associated with impaired trophoblast implantation and dysfunctional activity. In first-trimester trophoblast cell line, Sw.71, the addition of cortisol was shown to inhibit trophoblast cell invasion, thereby suppressing the implantation process (Smith et al., 2017; Kisanga et al., 2018). Cortisol exhibits a distinct endogenous circadian rhythm, modulated by sleep/wake cycles, dietary intake, and physical activity. Shift workers and night-shift nurses demonstrate significantly lower cortisol levels and display abnormal circadian rhythmicity due to circadian misalignment (Harris et al., 2010; Yang Z. et al., 2024). Cortisol demonstrates a strong correlation with peripheral circadian clock genes. *Bmal1*-knockout macaque monkeys exhibited significantly elevated cortisol concentrations accompanied by diminished oscillation amplitude, indicating disrupted glucocorticoid circadian regulation (Qiu et al., 2019). Exogenous cortisol administration significantly upregulated *Per1* expression in human peripheral blood mononuclear cells (PBMCs), while concurrently inducing phase shifts in the circadian oscillations of *Per2*, *Per3*, and *Bmal1* transcriptional patterns (Cuesta et al., 2015). Collectively, these findings suggest that cortisol is under circadian regulation and plays a regulatory role in embryonic implantation. Furthermore, the investigation of the circadian rhythm-cortisol-embryonic implantation axis and cortisol-mediated regulation of clock genes during implantation warrants further investigation to establish more direct mechanistic evidence.

### 3.5 Ghrelin- leptin

Ghrelin, an appetite-stimulating hormone produced by gastric P/D1 cells, and leptin, an appetite-suppressing hormone secreted by white adipocytes, have been demonstrated to participate in human endometrial decidualization processes (Cervero et al., 2004; Tawadros et al., 2007). Ghrelin demonstrates substantial upregulation during decidualization and facilitates human UESCs decidualization through activation of the growth hormone secretagogue receptor signaling pathway (Tanaka et al., 2003; Tawadros et al., 2007). Diet-induced obese murine models exhibit delayed decidualization and disrupted leptin signaling (Walewska et al., 2024). Exogenous leptin supplementation significantly enhanced embryo implantation efficiency in both *in vivo* and *in vitro* experimental systems through leptin receptor-mediated janus kinase (JAK)/signal transducer and activator of transcription (STAT) activation pathways (Yang et al., 2006; Barnes et al., 2020). In healthy adults under energy-balanced conditions, circulating ghrelin levels exhibit a 24-h oscillatory pattern synchronized with circadian rhythms (Cummings et al., 2002). *In vitro* experiments have also revealed that Ghrelin promotes decidualization since, the peptide enhances the production of insulin-like growth factor binding protein-1 (IGFBP-1) by human UESCs (Tawadros et al., 2007). Sleep deprivation and circadian misalignment contribute to obesity pathogenesis, primarily through metabolic dysregulation characterized by elevated ghrelin concentrations and suppressed leptin levels, thereby promoting positive energy intake (Wright, 2009; Qian et al., 2019). Genetic knockout models of *Bmal1*, *Clock*, *Per2*, or *Rev-erbs* exhibited elevated leptin levels (Turek et al., 2005; Yang et al., 2009; Kennaway et al., 2013; Adlanmerini et al., 2021). Leptin administration downregulated *Cry1* expression while concurrently upregulating *Rev-erbα* transcriptional activity (Vieira et al., 2012; Wei et al., 2021). In contrast, *Bmal1*-knockout mice displayed reduced ghrelin levels (Laermans et al., 2015). Accumulating evidence demonstrates that ghrelin and leptin exhibit robust bidirectional interactions with the circadian regulatory system. These metabolic hormones are rhythmically modulated by circadian oscillators to participate in embryo implantation processes.

### 3.6 Insulin

Insulin is secreted by pancreatic β-cells in response to fluctuations in blood glucose levels (Tokarz et al., 2018). During embryo implantation, insulin suppresses the production of IGFBP-1 in UESCs, which is recognized as a biochemical marker of decidualization. Consequently, hyperinsulinemic conditions are postulated to disrupt normal metabolic homeostasis in the endometrium, compromising implantation success through dysregulation of decidualization-related molecular pathways (Giudice et al., 1992). Hyperinsulinemia represents a hallmark metabolic aberration in polycystic ovary syndrome (PCOS). Notably, PCOS patients exhibit concomitant insulin resistance that downregulates glucose transporter 4 (GLUT4) expression, subsequently impairing glucose transporter activity. This metabolic dysfunction manifests as reduced cellular glucose uptake capacity and abnormal glucose metabolism homeostasis, ultimately contributing to aberrant endometrial differentiation and compromised embryo implantation competence (Zhai et al., 2012). Insulin signaling exhibits a close interplay with circadian rhythms. Shift workers demonstrate reduced insulin sensitivity. The SCN governs 24-h rhythmicity in blood glucose concentrations, with SCN-lesioned mice displaying abolished circadian glucose regulation (Fleur et al., 1999). Genetic ablation of core clock components, including *Bmal1*, *Clock*, or *Cry1/2*, induces hyperglycemia in murine models (Zhang et al., 2010; Reinke and Asher, 2019). Muscle-specific *Bmal1* knockout mice manifest impaired muscle insulin sensitivity (Harfmann et al., 2016). Notably, adenovirus-mediated *Cry1* overexpression enhances systemic insulin sensitivity in experimental animals (Zhang et al., 2010). Circadian regulation demonstrates a critical mechanistic connection with insulin homeostasis, with particular pathophysiological implications for insulin resistance. Emerging evidence implicates circadian disruption in compromised embryo implantation processes through its modulatory effects on insulin resistance pathways.

As summarized in Table 2, circadian rhythms regulate multiple implantation-critical factors—including BMP2/4, GDF10/15, COX2, LIF, IL-6, Cortisol, Ghrelin, Leptin and Insulin—through direct modulation of key processes such as embryo adhesion, trophoblast invasion, endometrial receptivity, and decidualization, thereby demonstrating their essential role in orchestrating successful embryo implantation.

**TABLE 2:** Circadian coordination of implantation factors in embryo implantation.

Factors		Produce	The regulatory role of circadian rhythm	Roles in implantation
Growth factors	BMP2/4	UESCs	Clock-controlled genes	Decidualization
	GDF10/15			
Prostaglandins	COX2			Adhesion
Cytokines	LIF	Macrophage	Circadian clock regulation	Receptivity
	IL-6			
Cortisol		Adrenal gland		Invasion
Ghrelin		Adipocytes		Decidualization
Leptin		Stomach		
Insulin		Pancreas		

### 3.7 Potential factor

It is well-established that sunlight exposure facilitates vitamin D synthesis, wherein ultraviolet B (290–320 nm, UVB) radiation converts cutaneous 7-dehydrocholesterol (7-DHC) into vitamin D3 through photochemical reactions. Vitamin D exerts pleiotropic physiological functions, with critical roles in reproductive physiology particularly in embryo implantation. Clinical evidence indicates vitamin D deficiency directly contributes to implantation failure (Halloran and Deluca, 1980). Notably, vitamin D supplementation in normally cycling mice significantly enhanced embryo implantation rates (Lee et al., 2024). The skin serves not only as the primary site of vitamin D synthesis but also as a key model system for circadian clock regulation, with multiple physiological skin processes exhibiting circadian rhythmicity. UVB irradiation was found to entrain rhythmic expression of *Bmal1* and *Per2* in human HaCaT keratinocytes (Lamnis et al., 2024). Emerging evidence further links vitamin D status to systemic circadian homeostasis: Vitamin D depletion induces hepatic clock gene dysregulation, characterized by downregulated *Bmal1*, *Clock*, *Per2*, and *Cry1/2* mRNA levels at ZT1, contrasted by paradoxical upregulation of these transcripts at ZT13 (Li R. et al., 2024). Conversely, vitamin D supplementation amplified the amplitude of *Per1*:luc circadian oscillations and enhanced rhythmic precision in human bone marrow stromal cells (BMSCs) (Hassan et al., 2017). While accumulating evidence suggests that vitamin D exhibits significant entrainment relationships with circadian clocks, the regulatory role of circadian rhythms in mediating vitamin D’s effects during embryo implantation warrants further investigation.

There is evidence demonstrating that female *Hoxa10* ^−/−^ mice experience infertility and implantation failure when wild-type embryos are transferred into them. Meanwhile, the expression of HOXA10 is remarkably enhanced during implantation, and specific inhibition of HOXA10 in the endometrium leads to a decrease in the number of implanted embryos. Besides its role in receptivity, HOXA10 also plays a significant part in endometrial decidualization. It is prominently expressed during decidualization. In 40% of the mice with HOXA10 null mutation, although implantation was successful, local hemorrhage at the implantation site, disordered embryos and empty decidua were observed. These results have also been validated *in vitro* (Benson et al., 1996; Lim et al., 1999; Modi and Godbole, 2009). In a research study carried out on hamsters, it was observed that a daily light exposure regimen with a longer light period (light/dark cycle of 16/8) led to a diminished expression level of HOXA10 and a greater frequency of uterine abnormalities when contrasted with the normal light exposure setting (light/dark cycle of 12/12). The identical outcomes were also manifested in mice that were exposed to continuous light (Das et al., 2022b; 2022a). Currently, there is a lack of reports regarding the specific mechanism by which particular clock genes act on HOXA10. However, based on existing reports and the promoting effect of MT on endometrial HOXA10, HOXA10 is clearly influenced by circadian rhythms (Guan et al., 2022).

Previous studies have clearly manifested that adhesion molecules are of crucial importance in the intimate and essential interaction between the blastocyst and the uterine luminal epithelial cells. Integrins, which are heterodimeric cell surface glycoprotein receptors composed of α and β subunits, serve as mediators for cell adhesion, cell migration, signal transduction, and gene expression (Hynes, 2002; Calderwood, 2004). The apical localization of integrin β3 has been thoroughly documented and recorded throughout the entire course of early pregnancy, accompanied by a significant increase precisely at the time of implantation in the mouse. During the critical moment of implantation, integrin β3 was observed to dissociate from focal adhesions. Simultaneously, integrin β3 demonstrated an augmented presence along the apical membrane of uterine luminal epithelial cells. This particular manifestation suggests that integrin β3 potentially has a substantial and influential role in the intricate process of embryo attachment (Kaneko et al., 2011b; 2011a). Integrin β5 demonstrates a circadian rhythm within retinal pigment epithelial cells and is modulated by *Bmal1* in human muscle tissue. In the case of mice, the expression of integrin β3 in platelets was diminished subsequent to the knockdown of *Rev-erbα* (Milićević et al., 2019; Shi et al., 2022). To date, the interaction between the circadian clock and integrin β3 remains unreported in uterus. Nevertheless, during embryo implantation, the pathological decrease in integrin β3 was reversed upon the addition of supplemental melatonin (Guan et al., 2022). Based on these observations, we hypothesized that integrin β3 may possess the potential to exhibit chronobiological effects.

In addition to vitamin D, HOXA10, and integrins, numerous potential factors regulated by circadian rhythms are involved in embryo implantation. Including P-selectin (Burrows et al., 1994; Qin and Deng, 2015), E-cadherins (Riethmacher et al., 1995; Li et al., 2018) and matrix metalloproteinases 9 (Bai et al., 2005; Li D. et al., 2024), While their roles in the embryo implantation process are well-established and circadian regulation has been evidenced, their specific mechanisms of influence during intrauterine implantation require further investigation.

## 4 Melatonin is a promising therapeutic candidate for addressing implantation failure caused by circadian rhythm disruptions

The function of clock-regulated MT in the realm of reproduction is currently under intensive investigation. As previously noted, MT exhibits a significant therapeutic efficacy in addressing implantation failure that arises from circadian rhythm disruptions. In this context, the pineal gland is the primary source of MT secretion, with a relatively higher output compared to the ovary. The MT synthesized within the mitochondria of oocytes and granulosa cells in the ovary remains unsecreted, whereas the MT synthesized by mitochondria in pineal cells is released into the third ventricle and cerebrospinal fluid and subsequently distributed throughout the organism. The significance of MT in embryo implantation is quite prominent. It plays a crucial part in tissue remodeling, angiogenesis, as well as in the suppression of inflammation during the process of embryo implantation (He et al., 2015). Our particular emphasis has been placed more on its role in modulating peripheral clock genes (Soliman et al., 2015; Ma et al., 2023). Within the mouse striatum, MT induces an upregulation in the expression of *Clock* and *Per1*. Moreover, when the pineal gland is surgically removed, the circadian rhythms of *Per1* and PER1 are disrupted. In the mouse tubercle, following MT1 knockout, the expression levels of *Bmal1*, *Clock*, *Per1*, and *Cry1* are observed to decline. In isolated rat adipocytes, MT serves to enhance the expression of *Bmal1*, *Clock*, *Per1*, and *Cry1*. In rats, it has also been demonstrated that MT modulates the circadian clock loop by modifying the rhythm of *Rev-erbα* (Von Gall et al., 2005; Agez et al., 2007; Alonso-Vale et al., 2008; Imbesi et al., 2009). MT not only governs the transcription/translation feedback loop of rhythm genes but also facilitates alterations in the firing rate of neurons within the SCN, thereby contributing to the stabilization of the body’s biological rhythm (Mauviard et al., 1991; Gillette and McArthur, 1995). Previous studies have demonstrated that MT rescues continuous light exposure-induced luteal insufficiency in mice, thereby promoting progesterone production (Li et al., 2023). Prolonged light exposure (18:6 light/dark cycle) significantly reduced embryo implantation rates in mice, and this adverse effect was reversed by MT supplementation (Zhang et al., 2017). Consequently, MT presents itself as a potentially favorable alternative for the treatment of implantation failure that is induced by circadian rhythm disruptions.

## 5 Conclusion

Implantation represents a crucial female reproductive process that is both centrally and peripherally modulated by circadian rhythms. Herein, we emphasize the significant role of circadian regulation in reproductive hormones, endometrial receptivity, and decidualization during embryo implantation. Generally speaking, while the overarching role of circadian rhythm in embryo implantation is relatively well-recognized, the specific underlying mechanisms still require further in-depth investigation. There exist several essential procedures within the process of embryo implantation, namely, blastocyst activation, positioning, adhesion, invasion, and decidualization. However, existing studies exhibit deficiencies in numerous aspects, and additional research data are essential to substantiate the clock regulation mechanism during embryo implantation. This is of great significance in order to expedite the resolution of the issue of abnormal embryo implantation attributable to circadian rhythm disorders.
